# 白细胞介素-1与肿瘤

**DOI:** 10.3779/j.issn.1009-3419.2010.12.12

**Published:** 2010-12-20

**Authors:** 骧 吴, 克 徐

**Affiliations:** 1 天津 300052，天津医科大学总医院中心实验室 Core Facility Center, Tianjin Medical University General Hospital; 2 天津市肺癌研究所，天津市肺癌转移与肿瘤微环境重点实验室 Tianjin Key Laboratory of Lung Cancer Metastasis and Tumor Microenviroment, Tianjin Lung Cancer Institute, Tianjin Medical University General Hospital, Tianjin 300052, China

炎症与感染会促进癌症的发生和发展^[1-5]^。临床资料和流行病学研究显示，慢性感染与炎症和癌症之间存在较强的相关性。例如，长期酗酒会引发肝脏和胰脏的炎症，进而导致这些器官癌症的发生机率增加；长期吸烟和接触石棉、硅石都会导致肺部的感染，进而使发生肺癌的可能性增加。最新的基于人类肿瘤和鼠类肿瘤模型的研究^[6-8]^结果表明，肿瘤微环境中的炎症反应是肿瘤发生与发展中的一个重要环节。将炎症与癌症联系起来的一个重要因素是活化的机体免疫细胞和癌细胞产生的细胞因子，它们可以促进肿瘤的生长和发展。巨噬细胞产生的白细胞介素-1β（interleukin-1β, IL -1β）可以引起炎症反应，还引起促炎基因的表达，如环氧化物酶-2（cyclooxygenase type 2, COX-2）、一氧化氮合成酶（inducible nitric oxide synthase, iNOS）、基质金属蛋白酶（matrix metalloproteinases, MMPs）等^[9]^。研究表明，肿瘤微环境中IL-1是很丰富的，主要由肿瘤微环境中的免疫细胞或恶性细胞产生，它影响恶性肿瘤的发展进程，如肿瘤的发生、发展及肿瘤的侵袭、转移。

## 1 IL-1α与IL-1β及其信号传导通路

IL-1的cDNA最早于1984年由Dinarello等克隆成功^[10]^。IL-1家族主要包括IL-1α和IL-1β。IL-1α和IL-1β与相同的受体结合，发挥相同的生物学功能，但是IL-1α与IL-1β又有着很大的区别。IL-1β只有分泌型具有活性，它的前体物没有活性，没有膜结合型IL-1β；而IL-1α主要是细胞结合型具有活性，包括细胞内前体（pro IL-1α）和膜结合型IL-1α，很少有分泌型。IL-1β在正常生理情况下不产生，它只有在炎性信号下分泌；而IL-1α在正常生理情况下存在于细胞质中和细胞膜上，在炎症发生时，它的表达水平升高。IL -1家族中还有一个成员——IL -1受体拮抗物（IL-1Ra），它与IL-1受体结合，但是不传导激活信号，是IL-1的生理抑制剂。

IL-1受体属于免疫球蛋白超家族，在许多种类的细胞上都有表达。IL-1受体Ⅰ（IL-1RI）（80 kDa）是信号传导受体，而IL -1受体Ⅱ（IL -1RII）（68 kDa）是一个假的目标，它的作用是减少多余的IL-1。IL-1与受体Ⅰ结合后，另一个蛋白，IL-1受体接受蛋白（IL-1RAcP）被募集，IL -1RI与IL -1RAcP组成的杂合二聚体触发IL -1的信号传导。二聚体首先激活了IL -1受体结合激酶（IL -1 receptor-associated kinase, IRAK），通过核转录因子NF-κB的介导，最终导致核基因的激活。与此相反，IL-1受体Ⅱ和IL-1受体拮抗物（IL-1Ra）不具备与IL-1RAcP结合，进而募集IRAK的能力。

## 2 IL-1的生物学作用

IL-1是一个多效性的细胞因子，它主要影响炎症和免疫反应，也调节机体其它生理功能，对疾病的发病机制有重要影响。在急性炎症疾病（如败血性休克）、慢性炎症疾病（如类风湿关节炎）和恶性肿瘤中IL-1水平均升高。

IL-1由巨噬细胞分泌，启动炎性反应^[11-13]^。IL-1α和IL- 1β不仅引起炎性反应，更重要的是它们诱导促炎基因的表达，其中最主要的有COX-2，可诱导的iNOS、MMPs、IL-6和其它趋化因子/细胞因子等。这些促炎基因的表达引起基质细胞和免疫细胞的快速激活，产生大量的前列腺素E2（prostaglandin E2, PGE2）、NO和细胞因子。IL-1还可以增强高亲和力粘附分子在内皮细胞、基质细胞和白细胞中的表达，包括整合素和免疫球蛋白超家族类粘附分子，并通过这一机制促进炎性细胞从血液到组织的浸润。IL -1β很容易在体液中被检测到，与IL -1α相比，IL-1β与炎症的联系更紧密。

IL-1对免疫功能也有调节作用^[11, 12]^。IL-1对机体内不同的细胞，如NK细胞、巨噬细胞、粒细胞等，及特殊的免疫活性细胞（包括T细胞和B细胞）的增殖、分化和功能有不同的强化作用。通过调控T细胞、抗原递呈细胞（antigen presenting cell, APC）的水平或调解微环境中细胞因子水平，IL-1调控CD4^+^、CD8^+^T细胞中Th1和Th2两个亚型的激活。

IL-1也被称为血细胞生成素-1（hemopoietin-1），是维持造血功能必需的造血因子，它可以诱导祖细胞上集落刺激因子（colony-stimulating factor, CSF）受体的表达，它还可以刺激基质细胞、白细胞和其它细胞中CSF和其它造血因子的表达和分泌^[14]^。IL-1对造血功能的影响有些不是直接的，而是通过激活骨髓基质细胞来介导的，在基质细胞中，IL-1刺激骨髓造血细胞因子的产生，还调节粘附分子的表达，其结果是加速血细胞从骨髓中的释放，加速它们向淋巴和外周组织的迁移。

## 3 IL-1对肿瘤生成的影响

研究表明，IL-1在肿瘤生成中起重要作用。Krelin研究小组^[15]^使用3-甲基胆蒽（3-MCA）诱导癌症的实验动物模型以分析IL-1在化学诱导肿瘤中的作用。用3-MCA处理正常对照小鼠和IL-1敲除小鼠，正常情况下，化学处理后3个月-5个月会发生纤维肉瘤。研究结果显示，在实验第110天，60%-70%的正常对照和IL-1α敲除小鼠（IL- 1α^-/-^）均已有肿瘤的发生，其余的动物也在晚些时间有肿瘤的发生；而IL-1β敲除小鼠（IL-1β^-/-^）和IL-1α与β双敲除小鼠（IL-1α/β^-/-^）中，3-MCA处理110天以后才开始有肿瘤的发生，且仅有部分动物发生，说明IL-1β的缺失抑制了3-MCA诱导的肿瘤生成。在IL-1受体拮抗物敲除小鼠（IL-1Ra^-/-^）中，IL-1水平无衰减，而且IL-1与受体之间的结合减弱了IL-1受体拮抗物的拮抗作用，肿瘤发展最迅速，所有动物在第110天都有肿瘤的发生。

肿瘤的生成是以靶细胞的遗传学改变作为起始事件的，发生突变的细胞不能正常凋亡，在刺激物刺激下增殖，积累更多的变异，最终导致明显的恶性肿瘤细胞的发展，进而形成肿瘤。IL-1参与了癌症发生的起始阶段-突变形成阶段，通过激活浸润的吞噬细胞或成纤维细胞等靶细胞的变异（transformation），产生使突变形成的活性氧中间体（Reactive oxygen intermediates, ROI）或一氧化氮中间体（nitric oxide intermediate, NOI），然后局部的IL-1会通过刺激前恶性细胞的增殖来进一步促进恶性化进程，广泛的增殖导致前恶性细胞变异的积累^[11-13]^。

研究表明，在化学致癌剂处理的部位，存在着炎性反应，有促炎因子的表达，如IL-1β、COX-2和少量的IL- 1α和TNFα，这样的炎症反应对肿瘤产生的过程有促进作用。联系慢性炎症与肿瘤生成的一个必需的因素是癌症前期细胞或炎性细胞中NF-κB的激活^[16-20]^。微环境中的促炎细胞因子，激活靶细胞中的NF-κB，这就使细胞不能凋亡，促进它们的恶性化。炎性细胞中的NF-κB的激活又会引起促炎细胞因子的表达，这些促炎因子加速了前恶性细胞的生长，并促使它们发展为明显的肿瘤。

*IL-1*基因组结构的改变、表达或修饰的改变对癌变过程有至关重要的作用。在放射线引起的急性髓性白血病（acute myeloid leukemia, AML）模型小鼠中，发现了2号染色体的重排，涉及*IL-1β*基因的重排和失调，它通过解除多功能造血干细胞增殖调控的机制来引发鼠白血病的发生^[21]^。Engels等^[22]^系统地研究了大量炎症相关基因与肺癌发生的危险性之间的相互关系，结果显示*IL-1α*和*IL-1β*基因的多态性与肺癌发生危险性的增高有关，尤其是在有大量吸烟史的患者中。Landvik等^[23]^对*IL1B*基因调控区的单核苷酸多态性（single nucleotide polymorphism, SNP）进行了研究，发现特定的IL1B单倍型可以增加*IL1B*基因表达，增加患非小细胞肺癌（non-small cell lung cancer, NSCLC）的危险性。

## 4 IL-1对肿瘤侵袭、转移的影响

IL-1可以增强已存在的肿瘤的侵袭性，主要是通过启动新血管生成、诱导炎性因子（如MMPs、Heparanase、趋化因子和整合素等）在恶性细胞或内皮细胞的表达，导致肿瘤扩散和转移^[24-26]^。在实验肿瘤模型和癌症患者中，局部增高的IL-1水平经常与肿瘤侵袭和预后不良相关联。为了阐明宿主IL-1在肿瘤转移中的作用，Voronov等^[27]^研究了B16黑素瘤细胞在IL-1β敲除小鼠和IL-1α敲除小鼠中的成瘤性。在IL-1β敲除小鼠中，在足掌内或静脉分别接种肿瘤细胞后，没有局部肿瘤的发生和肿瘤肺转移的发生，相反，在对照组正常C57BL/6小鼠中，可以观察到明显的肿瘤发展。他们的结果显示，微环境中的IL-1β对B16黑素瘤细胞在小鼠体内成瘤过程中的血管生成和肿瘤转移起重要作用，相比而言，IL-1α的作用则很小。Vidal-Vanaclocha等^[28]^发现在鼠B16模型中，微环境衍生的IL-1促进黑素瘤的肝转移，脾内注射重组IL-1β或IL-1的强诱导剂脂多糖（lipopolysaccharide, LPS）后，可以观察到肝转移的增加，当用IL-1Ra处理动物后转移减少，生存率上升。

基质由间质连结组织组成，包括成纤维细胞、炎性细胞（如淋巴细胞、巨噬细胞和中性粒细胞）。基质对于肿瘤生长、侵袭和新血管形成是必需的^[12, 13, 29]^。恶性肿瘤细胞以多种方式征服基质，以支撑它们侵袭的能力。研究表明，微环境中的基质细胞和恶性肿瘤细胞中，外源的重组的IL-1可以诱导生长因子、促侵袭因子的分泌，如MMPs；诱导促血管生成因子的分泌，如血管内皮生长因子（vascular endothelial growth factor, VEGF）、基础成纤维细胞生长因子（basic fibroblastgrowth fac-tor, bFGF）和ELR阳性CXC趋化因子，如IL-8和巨噬细胞趋化蛋白-1（monocyte chemotactic protein 1, MCP-1）^[9, 24]^。在人类肿瘤细胞系中进行的研究^[30]^表明，IL-1β通过NFкB和COX-2经典的炎症信号传导通路，上调功能性缺氧诱导因子（hypoxia-inducible factor, HIF-1α）的表达，进而增强肿瘤细胞分泌VEGF。通过促进新血管生成和其它恶性化进程，宿主和肿瘤细胞分泌的IL-1促进已存在的肿瘤的侵袭。

## 5 IL-1与人类肿瘤及临床靶向治疗

许多研究表明肿瘤细胞产生的IL-1增强人类肿瘤的转移。在慢性和急性髓性白血病、黑素瘤、前列腺癌、乳腺癌、胃癌、原发的星型细胞瘤中进行的大量研究结果显示，许多不同种类的人类肿瘤细胞产生IL-1α和β，表明IL-1参与了肿瘤的恶性化进程，这些结果有些是在已建立的肿瘤细胞系中得到的，有些是从新鲜的肿瘤样本中得到的。正常细胞只有在炎性信号或因子刺激下，才分泌细胞因子，肿瘤细胞可以在细胞因子或细菌产物的刺激下产生细胞因子，它本身还固有地分泌细胞因子。

IL-1可能充当着中心细胞因子的角色，不同的促新血管生成的刺激功能汇集于一身。因此，中和IL-1，就可以抑制下游一连串的有着促肿瘤新血管生成功能的促炎因子的产生。研究^[31, 32]^表明重组的IL-1Ra可以减小鼠肿瘤的体积，抑制肿瘤介导的新血管生成。IL-1Ra目前已被美国FDA批准用于临床，但主要用于类风湿关节炎患者的抗炎治疗，有很好的治疗效果。基于IL-1Ra可以中和分泌型IL-1β的作用，有些学者提出了将IL-1Ra用于癌症治疗的可能性^[33]^。IL-1Ra可以抑制肿瘤新血管生成、抑制肿瘤侵袭，在手术、化疗或放疗治疗原发肿瘤以后使用IL-1Ra可能是最有效的，可以阻止肿瘤的复发和转移。

## 6 结语

综上所述，IL-1参与了恶性肿瘤的发展过程。微环境中过表达的IL-1加速局部的炎性反应，进而加速肿瘤的发生。通过促进新血管生成和其它方面的恶性化，宿主或肿瘤细胞产生的IL-1促进肿瘤细胞的侵袭。IL-1作为重要的促炎因子，是将炎症与癌症联系起来的重要因素之一，对与炎症相关的其它细胞因子、代谢酶和催化酶与肿瘤相互关系的深入研究，将使我们对炎症与肿瘤的关系有更深入的了解、为人类更好的预防癌症、更有效的治疗癌症提供有力的理论基础。
